# Rational fluid management in today's ICU practice

**DOI:** 10.1186/cc11504

**Published:** 2013-03-12

**Authors:** Karsten Bartels, Robert H Thiele, Tong J Gan

**Affiliations:** 1 Department of Anesthesiology, Box 3094, Suite 5670B, Duke University Medical Center, Durham, NC 27710, USA; 2 Department of Anesthesiology, University of Virginia School of Medicine, PO Box 800710, Charlottesville, VA 22908-0710, USA

## Abstract

Intravenous fluid therapy has evolved significantly over time. From the initial report of the first intravenous administration of sodium-chloride-based solution to the development of goal-directed fluid therapy using novel dynamic indices, efforts have focused on improving patient outcomes. The goal of this review is to provide a brief overview of current concepts for intravenous fluid administration in the ICU. Results of recently published clinical trials suggesting harmful effects of starch-based solutions on critically ill patients are discussed. Concepts for goal-directed fluid therapy and new modalities for the assessment of fluid status as well as for the prediction of responsiveness to different interventions will continue to emerge. Advances in technology will have to be critically evaluated for their ability to improve outcomes in different clinical scenarios.

## Historical perspective

In 1832, following a suggestion by his mentor Dr William Brooke O'Shaugnessy, Dr Thomas Latta reported his physical examination findings after administrating a saline-based solution to a critically ill and dehydrated cholera patient, at a time when blood-letting was still the standard of care for this condition [1]:

Having inserted a tube into the basilic vein, cautiously-anxiously, I watched the effects; ounce after ounce was injected, but no visible change was produced. Still persevering, I thought she began to breathe less laboriously, soon the sharpened features, and sunken eye, and fallen jaw, pale and cold, bearing the manifest impress of death's signet, began to glow with returning animation; the pulse, which had long ceased, returned to the wrist; at first small and quick, by degrees it became more and more distinct, fuller, slower, and firmer, and in the short pace of half an hour, when six pints had been injected, she expressed in a firm voice that she was free from all uneasiness, actually became jocular, and fancied all she needed was a little sleep; her extremities were warm and every feature bore the aspect of comfort and health. [2]

This heretical yet highly successful intervention led to the achievement of a succession of important milestones, including the first use of intravenous albumin in 1834 by Dr John Mackintosh and the introduction of Ringer's solution in 1876 by Sidney Ringer [3], which was modified by Dr Alexis Hartmann to include lactate in 1876 [4]. Despite the accumulating evidence supporting the efficacy of intravenous fluid therapy for conditions that, in hindsight, would be labeled as some form of hypovolemia, the widespread use of intravenous fluid therapy would wait three-quarters of a century until the invention of the Rochester plastic needle - a needle-styletted plastic catheter - by the Mayo Clinic anesthesiology resident Dr David Massa in 1950 [5].

## Types of fluid

### Crystalloid solutions

Components of crystalloid solutions include inorganic ions such as sodium, chloride, potassium, magnesium, and calcium, as well as small organic substances such asglucose or lactate [6]. Examples of commonly used crystalloid solutions and their compositions are summarized in Table 1.

**Table 1:** Commonly used intravenous crystalloid solutions

Crystalloid solution	Components (mEq in 1,000 ml)	pH	Osmolarity (mOsmol/l)	Cost ($/1,000 ml)
Lactated Ringer's/Hartmann's solution	Sodium 130, chlorine 109, potassium 4, calcium 3, lactate 28	6 to 7.5	273	0.94
Ringer's acetate	Sodium 130, chlorine 112, potassium 5.4, calcium 0.9, magnesium 1, acetate 27	5.1 to 5.9	276	^a^
Normal saline	Sodium 154, chlorine 154	4.5 to 7	308	1.03
NormoSol-R, Plasma-Lyte A	Sodium 140, chlorine 98, potassium 5, magnesium 3, acetate 27, gluconate 23	7.4 (other pH formulations available)	295	2.21
Dextrose 5%, (variable concentrations available)	H_2_O, dextrose	3.2 to 6.5	252	0.96

Approximate sales price in the United States listed in US$ for commonly used unit sizes. ^a^Not readily available in the United States.

Relevant differences between these solutions include the potassium and calcium concentrations (which theoretically may affect their use in renal failure patients as well as their suitability for mixture with citrated blood products), chloride concentrations and strong ion difference (which ultimately affects the acid-base status of the recipient [7]), and cost.

One myth regarding crystalloid selection deserves further assessment - calcium-containing solutions are not absolutely contraindicated in the setting of transfusion. Most studies demonstrating clotting were based on citrate-phosphate-dextrose-adenine preserved packed red blood cells; newer preservatives, such as AS-3, contain less plasma and more citrate, and are much less likely to be overwhelmed by the small amount of calcium present in isotonic crystalloids [8].

### Colloid solutions

Colloid solutions consist of homogeneous, noncrystalline large molecules or ultramicroscopic particles (the internal phase) dispersed throughout another substance (the dispersion medium). Colloid solutions can be categorized as blood-derived, such as albumin, plasma protein fraction, and fresh frozen plasma, or as semisynthetic (hydroxyethylstarch (HES), dextrans, and gelatins) [6], several of which are described in Table 2. Base solutions for colloid preparations include normal and hypertonic saline, isotonic glucose, as well as other balanced solutions such as Ringer's lactate or Ringer's acetate.

**Table 2:** Commonly used intravenous crystalloid solutions

Colloid solution	Components (per liter)	Source	Cost ($)
Albumin 25%	12.5 g/50 ml human albumin	Human	46.42/50 ml
Plasma protein fraction 5%	50 g/l selected plasma proteins (88% albumin, 12% α-globulins and β-globulins, 1% γ-globulins), sodium 154 mEq, potassium 0.25 mEq, chlorine 100 mEq	Human	39.31/250 ml
Hydroxyethylstarch 130/0.4	Hydroxyethylstarch 130/0.4, 6% in 500 ml normal saline (other base solutions available)	Synthesized from amylopectin	47.13/500 ml
Hydroxyethylstarch 600/0.75	Hydroxyethylstarch 600/0.75, 6% in 500 ml normal saline (other base solutions are available)	Synthesized from amylopectin	15.55/500 ml
Gelatin 4%	40 g gelatinpolysuccinate	Bovine collagen	^a^
Dextran 40	10 g dextran 40, 5g dextrose	Biosynthesized from sucrose by *Leuconostoc *bacteria	20.55/500 ml

Approximate sales price in the United States listed in US$ for commonly used unit sizes. ^a^Not readily available in the United States.

The primary rationale for administering colloid solution in the setting of hypovolemia is the desire to expand plasma volume more effectively and extend the treatment duration. The larger solutes present in colloidal solutions are theoretically resistant to passage across the capillary membrane, and hence preserve intravascular oncotic pressure and prevent extravasation in accordance with the Starling equation. While this may work in theory, randomized controlled human trials comparing crystalloids with colloids suggest that the volumetric equivalence of colloids to crystalloids is not 1:3, as is classically taught, but somewhere between 1:1.3 and 1:2.1 [9].

## Colloid versus crystalloid

### *In vitro *and animal studies

Resuscitation of critically ill patients with colloid solutions has been suggested to improve oxygen delivery to the tissues. Initial evidence stems from a small experimental study in a rodent sepsis model, where resuscitation with HES (130 kDa) led to decreased leukocyte adhesion and a better maintained capillary integrity, as measured by macromolecular leakage, when compared with normal saline [10]. In an *in vitro *study into the effects of 25% albumin versus 6% HES (670 kDa) on endothelial inflammation, however, albumin seemed to show a modestly favorable profile as compared with HES [11].

In a sheep study of endotoxin-induced septic shock, resuscitation with larger molecular weight starch (HES 200/0.5) was found to cause a greater decrease in renal function and a higher degree of tubular disruption than therapy with HES 130/0.4 or crystalloid (Sterofundin^® ^ISO, B. Braun Melsungen, Germany) [12]. Since the study lasted only 12 hours, acute kidney disease could probably have worsened over time in either group. Deposition of HES into hepatocytes has been reported in patients with worsening hepatic dysfunction [13]. Although the complete mechanism for HES-associated renal dysfunction remains to be discovered, recent *in vitro *data suggest deposition of HES as vesicle-like structures into proximal tubular cells, as well as decreased cell culture survival with HES compared with incubation with crystalloids [14].

## Clinical studies

### Mortality in critically ill patients

The clinical relevance of the theoretical advantages of colloids (with regards to effects on the microvasculature) was questioned as early as the late 1970s [15]. Clinicians would have to wait almost 30 years, until the completion of the landmark Saline versus Albumin Fluid Evaluation (SAFE) trial, to begin to draw definitive conclusions. The SAFE trial compared the effects of 4% albumin versus normal saline in 6,997 critically ill patients [16] and found no difference in the primary outcome (all-cause mortality at 28 days). Subgroup analysis revealed a possible association between the use of albumin and increased mortality in patients with traumatic brain injury. It should be noted that this *post hoc *analysis was performed on only 492 patients or 7% of the original study population. In a follow-up report of 460 traumatic brain injury patients from the original SAFE trial, the association of albumin with higher mortality rates in this group persisted at 2 years following randomization [17].

The quality and power of previous trials on the safety of low molecular weight starches was questioned in a meta-analysis of 36 clinical studies (11 of which had previously been retracted) [18]. The more recent Scandinavian 6S trial compared low molecular HES (130/0.42) in a Ringer's acetate dispersion medium with Ringer's acetate without starch in the resuscitation of 804 septic ICU patients. This multicenter, randomized, and blinded clinical trial reported that use of up to 33 ml/kg of 6% HES (130/0.42) had a significantly higher risk of reaching the primary outcome, defined as death or need for renal replacement therapy at 90 days following randomization, in 51% of enrolled patients compared with 43% of enrolled patients receiving Ringer's acetate [19]. As only one patient in each group was receiving renal replacement therapy at 90 days, HES 130/42 increased the absolute risk of death at 90 days by 8 percentage points. Rates of severe bleeding were numerically higher in the HES group compared with crystalloid (10% vs. 6%), although this did not reach statistical significance (*P *= 0.09).

The Crystalloid versus Hydroxyethyl Starch Trial compared the blinded administration of up to 50 ml/kg body weight/day HES (130/0.4, Voluven; Fresenius Kabi AG, Bad Homburg, Germany) in normal saline versus normal saline alone in adult patients requiring fluid resuscitation following admission to an ICU [20]. The primary endpoint of all-cause mortality at 90 days was 17% in the normal saline group and 18% in the HES group. This difference did not reach statistical significance. Analysis of secondary endpoints revealed an association between HES use and acute kidney injury and a 21% relative risk increase for renal replacement therapy. Hepatic failure as well as increased use of blood products was also more common in the HES group, although a mild volume-sparing effect (90 ml and 246 ml difference in the volumes of study and nonstudy fluid, respectively, during first 4 days in ICU) was observed. In summary, HES (130/0.4) did not provide any substantial advantages to ICU patients requiring intravenous fluid resuscitation and the analysis of secondary endpoints confirmed an association of its use with renal injury.

### Acute kidney injury

Increasing degrees of oncocity in colloid solutions have been implicated in the incidence of acute kidney disease in a prospective nonrandomized cohort study of 1,013 ICU patients with shock. Both hyperoncotic semisynthetic colloids and hyperoncotic albumin were associated with increased incidences of renal events as compared with crystalloid solutions [21]. A prospective, non-randomized, cohort study of 346 patients who received HES, gelatin, or crystalloids for fluid resuscitation in the setting of sepsis showed acute kidney injury rates of 70%, 68%, and 47%, respectively [22]. In a retrospective study of 563 cardiac surgery patients, an independent association between the ad ministered dose of Pentastarch (250 kDa) and the development of acute kidney disease was made [23].

The clinical association of both high and low molecular weight starches with renal dysfunction has been confirmed in multiple clinical trials. In the randomized multicenter Volume Substitution and Insulin Therapy in Severe Sepsis trial, HES administration was associated with higher rates of acute kidney disease and renal replacement therapy than was administration of lactated Ringer's solution [24]. However, this was not the primary outcome of this trial. Most recently, the results of the Scandinavian 6S trial were made available: while the number of patients receiving renal replacement therapy at 90 days was no different, patients who were randomized to colloids were significantly more likely to require renal replacement therapy during their hospitalization (*P *= 0.04) [25].

### Hemodynamic differences and bleeding risk

Hartog's assertion that the hemodynamic advantages of colloids are overstated [9] has been supported by more recent data. In a randomized clinical trial of 196 sepsis patients receiving resuscitation, comparing HES 130/0.4 with normal saline, HES reduced the volume to reach hemodynamic stability only from 1.709 to 1.379 liters, but it had no effect on the cumulative fluid balance while in the ICU [26]. The assessment of safety profiles of currently used HES formulations had to be critically reevaluated, following the discovery of fraudulent research that possibly favored their use [27,28]. The association of HES and postoperative bleeding is well established. A decrease in factor VIII and von Willebrand factor have been linked to the older, large-molecular HES formulations [29] and similarly increased bleeding has been noted following cardiac surgery [30].

The current consensus statement of the European Society of Intensive Care Medicine already discourages the use of HES in patients with severe sepsis or acute kidney disease [31]. The results of both the recent Crystalloid versus Hydroxyethyl Starch Trial and the 6S-trial will probably result in a more narrow definition of its use in critically ill patients.

## Goal-directed fluid therapy

For decades, attempts at answering the question 'how much fluid do I give?' focused on the amount of fluid given (usually some arbitrary infusion rate with boluses as needed) and neglected the timing of fluid administration. Interpretation of the myriad of available liberal versus restrictive studies is complicated by a complete lack of standardized definitions of liberal and restrictive. In the early 2000s, several landmark papers suggested that there might be a more rational way to manage hemodynamics with fluid administration.

By manipulating hemodynamics using a complex formula designed to achieve specific targets for mean arterial pressure, urine output, and central venous oxygen saturation in septic patients, Rivers and colleagues showed that mortality could be improved by expanding one's hemodynamic goals beyond simply maintaining adequate blood pressure [32]. One year later, Gan and colleagues showed that a decrease in length of hospital stay and earlier return of bowel function was achieved using a protocol based on optimization of corrected flow time (an esophageal Doppler-derived index of preload) and stroke volume (also derived using esophageal Doppler monitoring) [33].

The clinical value of goal-directed fluid administration has also been demonstrated in other clinical settings and long-term beneficial effects in patients undergoing high-risk procedures have been suggested [34]. Limiting the total amounts of crystalloid infused was associated with decreased complications after major surgery in two groups that were randomized to a low rate or a high rate of crystalloid maintenance [35], suggesting that a fluid restrictive strategy in conjunction with goal-directed therapy might be beneficial. Not all data are supportive. In an earlier study by Gattinoni and colleagues, no difference in mortality in the ICU and at 6 months was detected in 762 critically ill patients randomized to three different hemodynamic goals (normal cardiac index, cardiac index >4.5 l/minute/m^2^, or normal mixed venous oxygen saturation ≥70%) [36]. A recent trial studying the effects of goal-directed intraoperative fluid therapy using esophageal Doppler monitoring failed to show a beneficial effect and actually found adverse effects in the intervention group. One should note that this study did not show a difference in the amount of fluid (colloid or crystalloid) administered to both groups [37]. Results of a randomized trial investigating mortality in 3,141 children with severe febrile illness and impaired perfusion in sub-Saharan Africa surprisingly showed higher mortality at 48 hours and at 4 weeks in the group that did not receive any fluid boluses compared with two groups resuscitated with albumin or saline [38]. These results challenge our understanding of the potential benefits of early fluid administration in septic shock and certainly merit further study in other relevant patient groups. However, since this study was performed in a resource-poor environment with limited intensive care and monitoring capabilities, conclusions are probably not directly translatable to clinical practice in other settings.

Overall, it appears that hemodynamic management protocols that focus on either preload or stroke volume optimization, as opposed to maintenance of an arbitrary threshold of blood pressure (or worse, continuous infusion of fluids at an unchanging rate), can improve outcomes. In a meta-analysis of 5,056 surgical patients randomized to tissue-perfusion-based hemodynamic protocols in 32 studies, mortality was reduced (pooled odds ratio = 0.67, 95% confidence interval = 0.55 to 0.82) [39]. Similarly, a meta-analysis in critically ill patients randomized to preemptive hemodynamic management (including 4,805 patients from 29 trials) also found a reduction in mortality (pooled odds ratio = 0.48, 95% confidence interval = 0.33 to 0.78) [40].

An exciting and relatively new series of hemodynamic endpoints that have firm grounding in cardiorespiratory physiology, but whose impact on clinical outcomes has not been fully discovered, has emerged. Dynamic indices attempt to predict the hemodynamic response to volume administration (that is, change in cardiac output after a standardized fluid bolus) and are based on the interaction between intrathoracic pressure changes and left ventricular end-diastolic volume and cardiac output. These new modalities seem to better answer the question 'what will happen to oxygen delivery if I administer fluids?' [41]. Common variants available in clinical practice include systolic pressure variation, pulse pressure variation, stroke volume variation, and the Pleth Variability Index. Systolic pressure variation, pulse pressure variation, and stroke volume variation can be determined via arterial blood pressure tracings. Stroke volume variation can also be obtained from minimally invasive methods, such as esophageal Doppler measurements, and non-invasive cardiac output monitoring using bioreactance technology - but other methods, such as low-frequency oscillations in the plethysmographic waveform (Pleth Variability Index), are also predictive of arterial blood pressure changes induced by mechanical ventilation, and have also been used to successfully predict fluid responsiveness [42]. To determine whether or not these new monitoring technologies will also lead to improved patient outcomes will require appropriately powered clinical trials in the future.

## Summary

Results of multiple smaller clinical trials and recent larger randomized controlled clinical trials advise against the use of both high and low molecular weight starch-based solutions in the care of critically ill patients, because they appear to increase morbidity and rates of renal dysfunction. No convincing data support their impact on improving outcomes. Based on the SAFE trial, albumin and crystalloids appear to have equal effects on mortality in critically ill patients, with the exception of patients with traumatic brain injury.

Goal-directed fluid therapy designed to optimize either stroke volume or preload has been validated in multiple patient groups and has the potential to improve meaningful clinical outcomes. Physicians should resist the blood-pressure-centric approach to hemodynamic management that has, for practical reasons, been the dominant paradigm for over a century, and should make every effort to utilize the currently available technology that can help them give fluids when they are needed, and to restrict them when they are not. Future studies should address both the ideal combination of monitoring devices as well as the choice of fluids in order to develop the best possible treatment strategy for specific clinical scenarios. In particular, stroke volume variation, arterial respiratory variation, and low-frequency oscillations in the photo-plethysmographic waveforms are attractive candidate endpoints that need to be further assessed.

A successful fluid management strategy needs to be incorporated into a multimodal interdisciplinary plan of care. We are reminded of this by Dr Latta's humbling closing of his first account of an initially successful resuscitation of a dehydrated cholera patient that fell short on these grounds:

This being my first case, I fancied my patient secure, and from my great need for a little repose, left her in charge of the hospital surgeon; but I had not been long gone, ere the vomiting and purging recurring, soon reduced her to her former state of debility. I was not apprised of the event, and she sunk in five and a half hours after I left her. As she had previously been of sound constitution, I have no doubt the case would have resulted in complete reaction, had the remedy, which already had produced such effect, been repeated. [2]

## Key messages

• HES appears to cause harm and should be avoided in the septic population and in patients at risk for kidney injury.

• The timing of fluid administration is just as important as (if not more important than) the amount given.

• Goal-directed fluid therapy designed to optimize either stroke volume or preload is well established in high-risk patient groups and should be considered in all critically ill patients.

• New modalities for assessment of dynamic indices offer non-invasive options to guide fluid therapy and assess the likely hemodynamic response to volume administration.

## Abbreviations

HES: hydroxyethylstarch; SAFE: Saline versus Albumin Fluid Evaluation.

## Competing interests

TJG has received research fundings from Fresenius Kabi, Inc.
