# Correction: Rational fluid management in today's ICU practice

**DOI:** 10.1186/cc12771

**Published:** 2013-06-26

**Authors:** Karsten Bartels, Robert H Thiele, Tong J Gan

**Affiliations:** 1 Department of Anesthesiology, Box 3094, Suite 5670B, Duke University Medical Center, Durham, NC 27710, USA; 2 Department of Anesthesiology, University of Virginia School of Medicine, PO Box 800710, Charlottesville, VA 22908-0710, USA

## Correction

Our recently published review article [1] contained a misprint. On page four under the sub-heading 'Goal-directed fluid therapy' and in the third paragraph, the sentence:

"Results of a randomized trial investigating mortality in 3,141 children with severe febrile illness and impaired perfusion in sub-Saharan Africa surprisingly showed higher mortality at 48 hours and at 4 weeks in the group that did not receive any fluid boluses compared with two groups resuscitated with albumin or saline 38."

should have instead read:

"Results of a randomized trial investigating mortality in 3,141 children with severe febrile illness and impaired perfusion in sub-Saharan Africa surprisingly showed *lower *mortality at 48 hours and at 4 weeks in the group that did not receive any fluid boluses compared with two groups resuscitated with albumin or saline 38."

The authors sincerely regret this error.

## Competing interests

TJG has received research funding from Fresenius Kabi and honoraria from Hospira and Baxter. KB and RHT declare that they have no competing interests.

## References

[B1] Bartels K Thiele R Gan T Rational fluid management in today's ICU practice Critical Care 2013 17 Suppl 1 S6 23514431 10.1186/cc11504 PMC3603466

